# The serum concentration and activity of DPP4 is positively related with the severity of hyperthyroidism in patients with Graves’ disease

**DOI:** 10.1080/07853890.2023.2226910

**Published:** 2023-06-23

**Authors:** Xiaona Chang, Xiaoyu Ding, Jiaxuan Wang, Qingyun Cai, Guang Wang, Jia Liu

**Affiliations:** Department of Endocrinology, Beijing Chao-yang Hospital, Capital Medical University, Beijing, China

**Keywords:** DPP4, concentration, activity, Graves’ disease, hyperthyroidism

## Abstract

**Objective:**

Graves’ disease (GD) is an organ-specific autoimmune disease. The production of anti-thyrotropin receptor antibodies (TRAb) is associated with a loss of immune tolerance. Dipeptidyl peptidase-4 (DPP-4) is expressed on multiple immune cells. This study aimed to investigate the relationship between serum concentration/activity of DPP4 and the severity of hyperthyroidism in GD patients.

**Methods:**

A total of 82 newly diagnosed drug-naive patients with GD hyperthyroidism, 20 patients with non-autoimmune thyrotoxicosis and 122 age- and sex- matched healthy controls were enrolled. The clinical parameters and serum concentration and activity of DPP4 were measured.

**Results:**

The GD group had increased serum concentration and activity of DPP4 than the healthy controls and patients with non-autoimmune thyrotoxicosis, while no significant difference was observed in the latter two groups. Multivariate linear regression indicated that the serum concentration/activity of DPP4 were positively associated with FT3, FT4 and TRAb levels in the GD patients. And the positive association between serum concentration/activity of DPP4 and TRAb was remained even after adjustment for confounding factors (all *p* < 0.05).

**Conclusions:**

The GD patients had significantly increased serum concentration/activity of DPP4. And the serum concentration/activity of DPP4 was positively associated with the severity of hyperthyroidism in GD patients.

**Key messages**
The activity and concentration of DPP4 in patients with Graves’ disease were higher than those in healthy controls.
There was a significant positive correlation between serum DPP4 concentration and TRAb levels in patients with Graves’ disease.
In patients with Graves ‘disease, serum DPP4 activity was positively correlated with TRAb levels.

## Introduction

Graves’ disease (GD), an organ-specific autoimmune disease, is the main cause of hyperthyroidism [1]. GD is characterized by overproduction of thyroid hormone, diffuse goitre, as well as positive test for anti-thyrotropin receptor antibodies (TRAb) [1]. The binding of TRAb to thyrotropic hormone receptor (TSHR) expressed on thyroid follicular cells leads to increased production and secretion of thyroid hormones, including thyroxine (T4) and triiodothyronine (T3), further leading to hyperthyroidism [1,2]. As a pathogenic autoantibody, the production of TRAb is associated with a loss of immune tolerance and dysfunction of immune cells [1,2].

Dipeptidyl peptidase-4 (DPP-4), also known as CD26, is commonly expressed on the surface of many cell membranes, but can also be found in circulation, resulting in the existence of DPP4 in both membrane-bound and soluble isoforms [3]. DPP4 is released from the membrane by a non-classical secretory mechanism through specific protein decomposition to produce a soluble form with similar enzyme activity [3]. DPP4 is broadly expressed on multiple cell types, including immune cells (T cells, B cells and monocytes), suggesting that the change in expression and activity of DPP-4 might be involved in the pathogenesis of autoimmune diseases [4,5]. Several previous studies showed that patients within type 1 diabetes had decreased DPP-4 expression of lymphocyte membrane but increased serum DPP-4 activity [6]. And the decreased serum concentration and activity of DPP-4 was observed in patients with multiple sclerosis [7]. Autoimmune thyroid diseases (AITDs) mainly include GD and Hashimoto’s thyroiditis [8]. A recent study found that patients with Hashimoto’s thyroiditis had decreased serum concentration and activity of DPP4 [9,10]. DPP4 inhibitors have been widely used as therapeutic agents for type 2 diabetes [11]. A recent study showed that in patients with GD and type 2 diabetes, DPP4 inhibitors administration was significantly associated with GD exacerbation, which suggested that DPP4 might play a critical role in the pathogenesis of GD [12]. However, the status of DPP4 in GD patients remained unknown. The present study aimed to investigate the relationship between serum concentration and activity of DPP4 and the severity of hyperthyroidism in GD patients.

## Materials and methods

### Study design and subjects

The present study consecutively recruited 82 newly diagnosed drug-naive patients with GD hyperthyroidism at the Endocrinology Department of Beijing Chao-Yang Hospital, Capital Medical University from September 2019 to December 2020. Moreover, we also enrolled 25 patients with iatrogenic thyrotoxicosis because of inappropriate levothyroxine dose (LT4 group), who had thyroid lobectomy because of papillary thyroid carcinoma (PTC) for at least two years and no suspected recurrence and metastasis during regular follow-up. And five of them were excluded because of previously diagnosed AITD or positivity of anti-thyroid peroxidase antibodies (TPOAb) and/or anti-thyroglobulin antibodies (TgAb) and/or TRAb (normal range for TPOAb or TgAb: 0.00 ∼ 60.0 IU/mL; normal range for TRAb: 0.00–1.75 IU/L). Meanwhile, 122 age- and sex- matched healthy controls were enrolled from the Physical Examination Centre of the same hospital. Hyperthyroidism was diagnosed when patients with decreased thyrotropic hormone (TSH) levels and increased levels of serum free T3 (FT3) and free T4 (FT4) levels (normal range: FT3: 2.63–5.71 pmol/L; FT4: 9.10–19.24 pmol/L; TSH: 0.35–4.94 mIU/L) [13]. GD was established when typical clinical presentation, positivity of TRAb and thyroid enlargement with abundant flow signals were observed [13]. The healthy control subjects were included when they had normal thyroid function, negative thyroid autoantibody (TPOAb, TgAb and TRAb) and normal thyroid ultrasound. No participants had a history of systemic inflammatory disease, infectious diseases, hypertension, diabetes, cardiovascular disease, liver and renal function impairment, or cancer. Moreover, participants who were pregnant or possibly pregnant or ingested agents known to influence thyroid function were also excluded.

This study was complied with the principles of the Declaration of Helsinki and approved by the Ethics Committee of Beijing Chao-Yang Hospital, Capital Medical University (application number: 2018-K330). Witten informed consent was obtained from each participant.

### Measurements of clinical parameters

The same trained group measured height and weight of each participant and collected their medical history. Height and weight were measured to the nearest 0.1 cm and 0.1 kg, respectively. BMI was calculated as weight in kilograms divided by height in metres squared. Thyroid function, including FT3, FT4 and TSH, were measured by electrochemiluminescence immunoassay with a functional sensitivity of 0.77 pmol/L for FT3, 1.3 pmol/L for FT4 and 0.005 mU/L for TSH (Dimension Vista, Siemens Healthcare Diagnostics, Germany). TPOAb and TgAb were detected by chemiluminescent immunoassay, and TRAbs were evaluated by electrochemiluminescence immunoassay (Dimension Vista, Siemens Healthcare Diagnostics, Germany). White blood cell (WBC), neutrophil, and lymphocyte counts were evaluated using a Beckman-Coulter Ac. T5Diff haematology analyser (Beckman-Coulter, Fullerton, CA, USA). Fasting plasma glucose (FBG) was measured by glucose oxidase method (Hitachi 747, Roche Diagnostics, Germany). Alanine aminotransferase (ALT) and aspartate aminotransferase (AST) were measured *via* velocity method (Hitachi 747, Roche Diagnostics, Germany). Fasting plasma insulin (FINS) was measured using the chemiluminescence method (Dimension Vista, Siemens Healthcare Diagnostics, Germany). Haemoglobin A1c (HbA1c) was estimated by high-performance liquid chromatography using the HLC-723G7 analyser (Tosoh Corporation, Tokyo, Japan). The thyroid ultrasound was assessed by a well-trained ultrasound physician. Homeostasis model assessment of insulin resistance (HOMA-IR) was calculated according to the following formula: HOMA-IR = FINS (mIU/L) × FBG (mmol/L)/22.5 [14].

### Measurements of serum DPP4 concentration and activity

In accordance with the manufacturer’s instructions, serum DPP4 concentration was detected by enzyme-linked immunosorbent assay (ELISA) (#DC260B, R&D, USA). In addition, serum DPP4 activity was measured by a commercial DPP4 activity assay kit (#MAK088, Sigma, Systems, USA). According to the instructions, DPP4 cleaves a non-fluorescent substrate, H-Gly-Pro-AMC, to release a fluorescent product, 7-Amino-4-Methyl Coumarin (AMC) (λ_ex_ = 360/λ_em_ = 460 nm). One unit of DPP4 is the amount of enzyme that will hydrolyse the DPP4 substrate to yield 1.0 μmole of AMC per minute at 37 °C.

### Statistical analysis

All statistical analyses were performed with SPSS 26.0 (SPSS, Chicago, IL, USA). The distribution of continuous data was evaluated using the Kolmogorov–Smirnov test. Normally distributed data were expressed as mean ± standard deviation. Because TSH, TRAb, TPOAb, TgAb, ALT, AST, FINS and HOMA-IR did not follow a normal distribution, the values were given as medians and upper and lower quartiles. The differences among the three groups (the control, LT4 and GD groups) at baseline were analysed by ANOVA test or Kruskal–Wallis H test followed by Bonferroni post hoc tests. The proportions were analysed using chi-squared tests. Correlation analyses were performed using Pearson and Spearman correlations. Multivariate regression analysis was performed to assess the relationship between serum concentration and enzymatic activity of DPP4 and relevant variables, and variance inflation factor (VIF) was used to identify multicollinearity. Statistical significance was considered with two-tailed analyses as *p* < .05.

## Results

### Baseline characteristics of control and GD groups

Table 1 presents the baseline characteristics of the control, LT4 and GD groups. Age and gender were comparable in the three groups. A significant difference was presented for BMI, FT3, FT4, TSH, TRAb, TPOAb, TgAb, neutrophil, lymphocyte, ALT, AST, FINS, HOMA-IR and the concentration and activity of DPP4 among the three groups (BMI, FT3, FT4, TSH, TRAb, TPOAb, TgAb, neutrophil, ALT, AST, DPP4 concentration and DPP4 activity: *p* < .01; lymphocyte, FINS and HOMA-IR: *p* < .05). There was no significant difference in the levels of WBC, FBG and HbA1c among the three groups.

**Table 1.** The baseline characteristics of the control, LT4 and GD groups.

Parameters	Control group (*n* = 122)	LT4 group (*n* = 20)	GD group (*n* = 82)	*p*
Age, years	41.4 ± 11.7	40.4 ± 11.1	39.6 ± 13.0	.293
Gender, M/F, n	28/94	4/16	19/63	.486
BMI, kg/m^2^	24.17 ± 4.03	22.95 ± 3.47	21.75 ± 3.42**	.000
FT3, pmol/L	4.98 ± 0.55	11.56 ± 6.06**	23.11 ± 7.72**^##^	.000
FT4, pmol/L	16.34 ± 2.07	36.43 ± 20.27**	68.49 ± 31.50**^##^	.000
TSH, μIU/mL	1.87 (1.22–2.25)	0.001 (0.01–0.03)**	0.01 (0.00–0.01)**	.000
TRAb, IU/L	0.60 (0.00–1.30)	0.80 (0.80–1.07)	9.41 (5.75–20.98)**^##^	.000
TPOAb, IU/mL	31.00 (28.00–49.62)	34.40 (28.00–50.20)	954.85(58.67–2937.20)**^##^	.000
TgAb, IU/mL	16.20 (15.00–22.25)	25.50 (15.00–49.00)	104.25 (30.02–250.75)**^##^	.000
WBC, 10^9^ /L	6.20 ± 1.72	6.06 ± 1.99	5.79 ± 1.54	.297
Neutrophil, 10^9^ /L	3.65 ± 1.32	3.81 ± 1.80	2.98 ± 1.13**^##^	.002
Lymphocyte, 10^9^ /L	2.01 ± 0.58	1.73 ± 0.37	2.20 ± 0.73^##^	.021
ALT, U/L	19.0 (14.0–27.0)	21.0 (11.0–39.0)	37.0 (22.5–48.5)**^#^	.000
AST, U/L	20.0 (17.0–24.0)	20.0 (15.0–28.0)	38.0 (21.5–44.0)**^##^	.000
FBG, mmol/L	5.10 ± 0.87	5.12 ± 0.74	5.24 ± 0.66	.225
FINS, mIU/L	9.30 (6.40–13.40)	8.35 (6.00–13.07)	12.10 (7.65–16.05)*^#^	.044
HOMA-IR	1.98 (1.38–2.99)	2.01 (1.31–3.26)	2.91 (1.81–3.64)**	.034
HbA1c, %	5.47 ± 0.39	5.48 ± 0.36	5.50 ± 0.42	.710
DPP4 concentration, ng/mL	576.73 ± 147.18	504.51 ± 134.79	739.28 ± 202.77**^##^	.000
DPP4 activity, microunits/mL	1217.64 ± 549.70	1357.78 ± 513.05	1775.08 ± 632.28**^##^	.000

*Notes:* Data are means ± SD unless indicated otherwise. TSH, TRAb, TPOAb, TgAb, ALT, AST, FINS and HOMA-IR are shown as median, upper and lower quartiles. M: males; F: females; BMI: body mass index; FT3: free triiodothyronine; FT4: free thyroxine; TRAb: anti-thyrotropin receptor antibodies; TPOAb: antithyroid peroxidase antibodies; TgAb: antithyroglobulin antibodies; WBC: white blood cell; ALT: alanine aminotransferase; AST: aspartate aminotransferase; FBG: fasting plasma glucose; FINS: fasting insulin; HOMA-IR: homeostasis model assessment of insulin resistance; HbA1c: haemoglobin A1c; DPP-4: dipeptidyl peptidase-4; LT4: levothyroxine; GD: Graves’ disease. Compared with the control group, *p < .05, **p < .01. Compared with the LT4 group, ^#^*p* < .05, ^##^*p* < .01.

**Figure 1.**
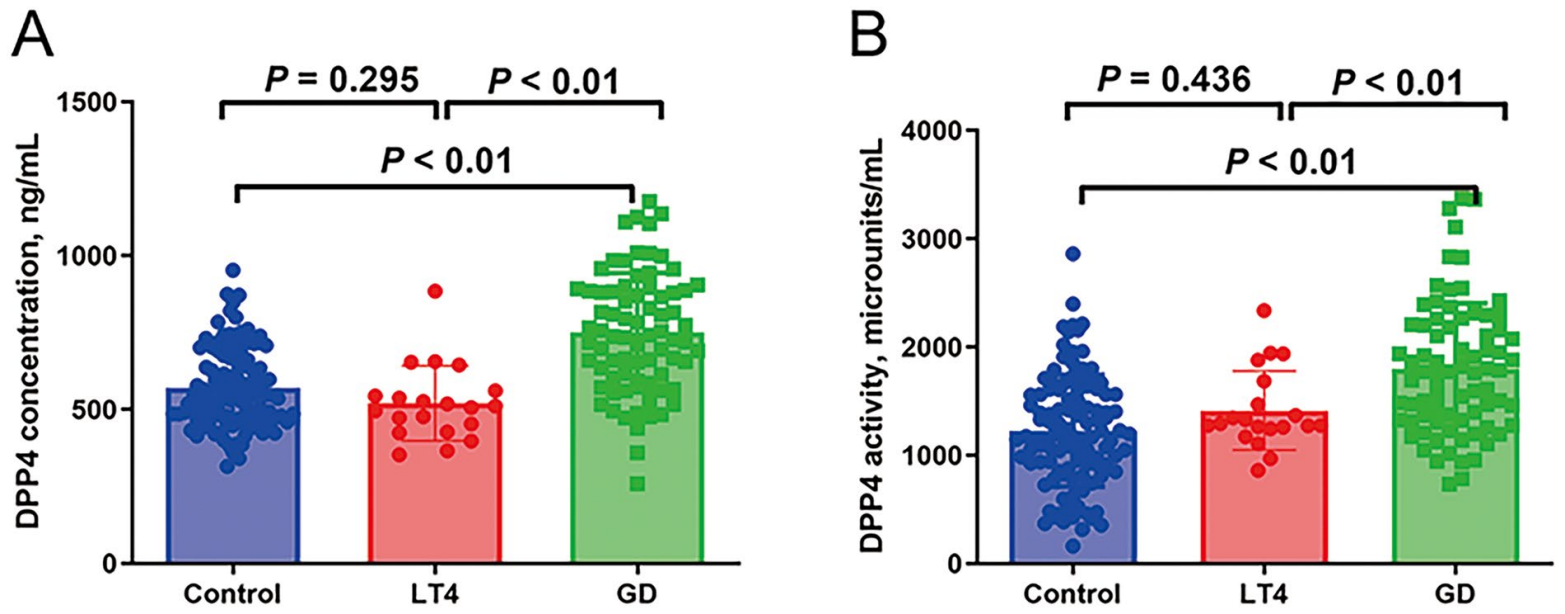
The DPP4 concentration (a)/activity (B) of the control, LT4 and GD groups. LT4: levothyroxine; GD: Graves’ disease; NS: no significance.

We next performed post hoc analyses and found that both the LT4 and GD groups had significantly increased levels of FT3 and FT4 and decreased TSH levels when compared with the control group (Table 1). The significant differences in BMI, TRAb, TPOAb, TgAb, neutrophil, ALT, AST, FINS and HOMA-IR were observed when comparing the GD group with the control group (Table 1). The GD group had increased FT3, FT4, TRAb, TPOAb, TgAb, lymphocyte, ALT, AST and FINS levels and decreased neutrophil count, as compared to the LT4 group (Table 1). Interestingly, the GD group had significantly higher concentration and activity of DPP4 than the control and LT4 groups, while no significant difference in concentration and activity of DPP4 was observed in the latter two groups (Table 1 and Figure 1).

### The correlation between serum concentration/activity of DPP4 and clinical parameters in the GD patients

Bivariate correlation analysis was used to investigate the correlation between serum concentration/activity of DPP4 and clinical parameters in the GD patients. The serum DPP4 concentration were positively associated with FT3, FT4, TRAb and lymphocyte levels and negatively associated with age and TSH (FT3: *r* = .469, *p* < .01; FT4: *r* = .486, *p* <.01; TRAb: *r* = .402, *p* < .01; lymphocyte: *r* = .231, *p* < .05; age: *r* = −.250, *p* <.05; TSH: *r* = −.229, *p* < .05; Table 2). There was no significant association between serum DPP4 concentration and other parameters, including BMI, TPOAb, TgAb, WBC, neutrophil, ALT, AST, FBG, FINS, HOMA-IR and HbA1c levels (Table 2).

**Table 2.** Correlation analysis between serum concentration/activity of DPP4 and clinical parameters in the GD patients.

	DPP4 concentration		DPP4 activity	
	*r*	*p*	*R*	*p*
Age	−.250	.028	−.109	.296
BMI	−.025	.827	−.178	.087
FT3	.469	.000	.343	.001
FT4	.486	.000	.398	.000
TSH	−.229	.027	−.297	.004
TRAb	.402	.000	.432	.000
TPOAb	−.101	.385	.012	.909
TgAb	−.080	.491	.089	.401
WBC	−.067	.532	−.010	.926
Neutrophil	−.087	.417	−.198	.061
Lymphocyte	.231	.029	.252	.017
ALT	.052	.618	.115	.269
AST	.185	.076	.173	.095
FBG	−.104	.373	−.093	.387
FINS	.034	.768	−.048	.679
HOMA-IR	−.007	.955	−.048	.687
HbA1c	.168	.225	.143	.153

*Note:* BMI: body mass index; FT3: free triiodothyronine; FT4: free thyroxine; TRAb: anti-thyrotropin receptor antibodies; TPOAb: antithyroid peroxidase antibodies; TgAb: antithyroglobulin antibodies; WBC: white blood cell; ALT: alanine aminotransferase; AST: aspartate aminotransferase; FBG: fasting plasma glucose; FINS: fasting insulin; HOMA-IR: homeostasis model assessment of insulin resistance; HbA1c: haemoglobin A1c; DPP-4: dipeptidyl peptidase-4; GD: Graves’ disease.

In the GD group, serum DPP4 activity were positively associated with FT3, FT4, TRAb and lymphocyte levels and negatively associated with TSH (FT3: *r* = .343, *p* = .01; FT4: *r* = .398, *p* <.01; TRAb: *r* = .432, *p* < .01; lymphocyte: *r* = .252, *p* < .05; TSH: *r* = −.297, *p* < .01; Table 2). There was no significant association between serum DPP4 activity and other parameters, including age, BMI, TPOAb, TgAb, WBC, neutrophil, ALT, AST, FBG, FINS, HOMA-IR and HbA1c levels (Table 2).

### Multivariate linear regression for the association of serum concentration/activity of DPP4 with thyroid function and TRAb

In model 1, serum concentration/activity of DPP4 were significantly associated with FT3, FT4, TSH and TRAb levels without adjustment of potential confounders (Tables 3 and 4). In model 2, with adjustment for age, gender and BMI, the association between serum concentration/activity of DPP4 and FT3, FT4 and TRAb levels was remained (Tables 3 and 4). In model 3, with further adjustment with age, gender, BMI, FT3, lymphocyte count, AST and HOMA-IR, we found that serum concentration/activity of DPP4 remained positively and independently associated with TRAb in the GD patients (DPP4 concentration: *β* = 0.265, *p* < .05; DPP4 activity: *β* = 0.325, *p* < .01). The regression equation held and fit well (DPP4 concentration: *F* = 3.881, *p* < .01; DPP4 activity: *F* = 3.852, *p* < .01), and the model waived the risk of multicollinearity (the highest VIF was 1.905) (Supplementary Table 1).

**Table 3.** Multivariate linear regression for the association of serum DPP4 concentration with thyroid function and TRAb in the GD group.

	Model 1		Model 2		Model 3	
Variables	Standardized *β* (95% CI)	*p*	Standardized *β* (95% CI)	*p*	Standardized *β* (95% CI)	*P*
FT3	0.451 (0.266, 0.636)	<.001	0.436 (0.243, 0.629)	.001	_	_
FT4	0.475 (0.293, 0.658)	<.001	0.467 (0.273, 0.660)	<.001	_	_
TSH	−0.229 (−0.430, −0.027)	.026	−0.217 (−0.423, −0.012)	.038	_	_
TRAb	0.524 (0.348, 0.700)	<.001	0.436 (0.246, 0.625)	<.001	0.265 (0.026, 0.504)	.030

*Note:* Model 1 unadjusted; Model 2 adjusted for age, sex and BMI; Model 3 adjusted for age, gender, BMI, FT3, lymphocyte count, AST and HOMA-IR. BMI: body mass index; FT3: free triiodothyronine; FT4: free thyroxine; TRAb: anti-thyrotropin receptor antibodies; AST: aspartate aminotransferase; HOMA-IR: homeostasis model assessment of insulin resistance; DPP-4: dipeptidyl peptidase-4; GD: Graves’ disease.

**Table 4.** Multivariate linear regression for the association of DPP4 activity with thyroid function and TRAb in the GD group.

	Model 1		Model 2		Model 3	
Variables	Standardized *β* (95% CI)	*p*	Standardized *β* (95% CI)	*p*	Standardized *β* (95% CI)	*P*
FT3	0.365 (0.172, 0.558)	<.001	0.356 (0.158, 0.554)	.001	_	_
FT4	0.433 (0.247, 0.620)	<.001	0.421 (0.225, 0.617)	<.001	_	_
TSH	−0.218 (−0.420, −0.016)	.035	−0.195 (-14.841, −0.351)	.061	_	_
TRAb	0.433 (0.262, 0.632)	<.001	0.428 (0.239, 0.616)	<.001	0.325 (0.086, 0.564)	.008

*Note:* Model 1 unadjusted; Model 2 adjusted for age, sex and BMI; Model 3 adjusted for age, gender, BMI, FT3, lymphocyte count, AST and HOMA-IR. BMI: body mass index; FT3: free triiodothyronine; FT4: free thyroxine; TRAb: anti-thyrotropin receptor antibodies; AST: aspartate aminotransferase; HOMA-IR: homeostasis model assessment of insulin resistance; DPP-4: dipeptidyl peptidase-4; GD: Graves’ disease.

## Discussion

The present study showed that the GD patients had significantly increased serum concentration and activity of DPP4 than the healthy controls and patients with non-autoimmune thyrotoxicosis. Multivariate linear regression analysis indicated that the serum concentration and activity of DPP4 were positively associated with FT3, FT4 and TRAb levels in the GD patients after adjusted for age, gender and BMI. And the positive association between serum concentration/activity of DPP4 and TRAb was remained even adjustment for age, gender, BMI, FT3, lymphocyte count, AST and HOMA-IR.

DPP-4, expressed on the surface of immune cells, is a multifunctional molecule involved in the pathophysiology of autoimmune diseases [4,5]. Many previous studies had shown that the serum concentration and activity of DPP4 changed in patients with autoimmune diseases, such as type 1 diabetes and multiple sclerosis [6,7]. There was the different trend in DPP4 activity and concentration between different autoimmune diseases. For example, sometimes DDP4 is high (Graves’ disease or type 1 diabetes), sometimes is low (autoimmune thyroiditis, rheumatoid arthritis). Maybe because shedding of DPP4 from different tissues seems to be varied at different disease conditions [3]. The present study investigated serum concentration and activity of DPP4 in GD patients and found that the GD patients had significantly increased serum concentration and activity of DPP4 than the healthy controls and patients with non-autoimmune thyrotoxicosis. However, a recent study observed decreased serum DPP4 levels in patients with Graves’ disease or Graves’ ophthalmopathy (GO) [15], which is inconsistent with our findings. The possible reasons for discrepant results are as follows: firstly, the baseline characteristics of subjects should be considered, such as BMI. Previous studies have demonstrated that circulating DPP4 levels are associated with BMI [16], and the effect of BMI on DPP4 may influence the serum DPP4 concentration and activity. Secondly, lymphocyte count was not included in their study, and the lymphocyte was associated with DPP4 levels and activity [3], which may affect the result. In addition, the ELISA results indicated that protein expression levels of DPP4 were ­significantly upregulated in thyroid-associated ophthalmopathy (TAO) patients compared with healthy controls [17]. This research supported our result. TRAB, the pathogenic autoantibody of GD, promotes production and secretion of thyroid hormones *via* binding with TSHR of thyroid follicular cells [1,2]. The present study indicated that the serum concentration and activity of DPP4 were positively associated with FT3, FT4 and TRAb levels in the GD patients after adjusted for age, gender and BMI. Interestingly, the positive association between serum concentration/activity of DPP4 and TRAb was still remained even adjustment for age, gender, BMI, FT3, lymphocyte count, AST and HOMA-IR. Therefore, the results suggested that the serum concentration and activity of DPP4 was increased and positively related with the severity of hyperthyroidism in GD patients.

However, we found the ALT and AST levels were significantly elevated in the GD patients comparing with the control group and LT4 group. It was consistent with the previous conclusion [18], which was liver function tests are frequently abnormal in patients with newly diagnosed thyrotoxicosis/hyperthyroidism. The underlying mechanism behind this seems to be the increased oxygen consumption consequent to the enhanced metabolic rate. This results in a relative hypoxia in the perivenular region, leading in turn to apoptosis and oxidative stress [18]. Additionally, the patients in GD group had the lower BMI, and this also attributed to the enhanced metabolic rate in GD.

The pathogenesis of GD is involved in the production of TRAb caused by the loss of immune tolerance and dysfunction of immune cells [1,2]. Histopathological features of GD patients included diffuse thyroid follicular cell hyperplasia and lymphocytic infiltration [19]. And the infiltrated lymphocytes are the main source of TRAb [20]. Besides, lymphocytes in bone marrow and lymph nodes sites had been demonstrated as extrathyroidal source of TRAb [21]. Meanwhile, lymphocytes are also the main sources of DPP4 [22]. Several previous studies have shown that DPP4 involves in the development, maturation, differentiation and activation of T and B lymphocytes and participates in immune regulation [23,24]. That leads on to the question: whether increased concentration/activity of DPP4 contributed to the development of GD? A recent human study showed that DPP4 inhibitors administration was significantly associated with GD exacerbation in patients with GD and type 2 diabetes [12]. Similarly, the association between DPP-4 inhibitors administration and increase risk of autoimmune diseases, including inflammatory bowel disease [25] and Hashimoto’s thyroiditis [26], was reported in some cohort studies. Moreover, DPP4 gene deletion caused increased T cell proliferation and type 1 cytokine production in mice [27]. In NOD mice, long-term inhibition of DPP-4 decreased the infiltration of CD4 + T cells in islets, alleviated insulitis and reduced serum levels of IL-1β and IL-12 [28]. When comparing the results from human and mice, it is important to consider that DPP4 expression in blood is different between humans and mice. In humans, DPP4 is mainly expressed by T cells, whereas in mice, DPP4 is also expressed by dendritic cells, B cells and NK cells [3]. Therefore, these results suggested that DPP4 might have a negative regulatory effect in autoimmunity and increased concentration and activity of DPP4 might be a compensatory response for protecting against immunologic abnormalities in GD patients.

The present study has several limitations. Firstly, the present study was a cross-sectional study, so we could not conclude a causal interpretation. Secondly, because the present study was a single-centre study with a relatively small sample size, especially in LT4 group, it might have some confounders to influence the results. Therefore, the generality of the results was restricted. Further prospective studies with a large sample size were needed to confirm the results of the present study. Thirdly, a better control group would have been non-autoimmune hyperthyroidism (for example toxic nodular goitre) since it would have been much similar to GD patients, except for the lack of autoimmunity. Regretfully, we didn’t include this population. Finally, we only measured the concentration and activity of DPP4 in serum of GD patients, LT4 over-treated people and healthy controls. Further studies are required to investigate the concentration, activity and the shedding process of DPP4 in thyroid and immune cells in GD patients.

In conclusion, the present study showed that the GD patients had significantly increased serum concentration and activity of DPP4. And increased serum concentration and activity of DPP4 was positively related with the severity of hyperthyroidism in GD patients. These findings might help to further understand the pathogenesis and immunoregulation mechanism in GD.

## Supplementary Material

Supplemental Material

## Data Availability

All data of this study are available from the corresponding author Jia Liu for reasonable request.
