# Supplementary material for: The serum concentration and activity of DPP4 is positively related with the severity of hyperthyroidism in patients with Graves’ disease
Source: Ann Med. 2023 Jun 23;55(1):2226910. doi: 10.1080/07853890.2023.2226910 (PMC10291917; doi:10.1080/07853890.2023.2226910)
Supplement: Supplemental Material [file IANN_A_2226910_SM1121.docx]

**Supplementary Table 1.** Multicollinearity test of multivariate linear regression for the association of serum DPP4 concentration/activity with thyroid function and TRAb in the GD group

|  | VIF |
| --- | --- |
| Age | 1.180 |
| Gender | 1.062 |
| BMI | 1.087 |
| FT3 | 1.905 |
| Lymphocyte count | 1.252 |
| HOMA-IR | 1.128 |
| AST | 1.182 |
| TRAb | 1.677 |

BMI: body mass index; FT3: free triiodothyronine; HOMA-IR: homeostasis model assessment of insulin resistance; AST: aspartate aminotransferase; TRAb: anti-thyrotropin receptor antibodies; DPP-4: dipeptidyl peptidase-4; GD: Graves' disease.
